# Supplementary material for: Facilitating the natural semi-drying of oily sludge by changing the form of water
Source: PLoS One. 2021 Jan 14;16(1):e0245430. doi: 10.1371/journal.pone.0245430 (PMC7808663; doi:10.1371/journal.pone.0245430)
Supplement: Attachment — Submitted filename: Manuscript Review - Liu (2020).pdf [file pone.0245430.s002.pdf]

Manuscript Review: Promoting the natural semi-drying of oily sludge by changing the form of water (Liu et al., 2020)

Summary

This research addresses the issue of disposal of oily sludge, a common waste of the petroleum industry. These residues contain large amounts of water, thus increasing the volume and mass of material for disposal. Reducing the water content reduces the amount of waste and thus the economic burden of its disposal.

Authors use an orthogonal experimental method to determine the ideal mixture of chemical reagents (emulsifiers, oxidants, cementitious materials and "other reagents") to use as an amendment to oily sludge to reduce drying times. The authors then carry out experiments to examine how temperature, exposure time and amendment concentration impact oily sludge drying time.

They show that chemical amendments can dramatically decrease the time needed for these wastes to reach a semi-dry (40-50% moisture content) state and thereby reduce the economic burden of oily sludge disposal. The novel aspect of this research is the application of chemicals to aid in reaching a semi-dry state. Research to date has focused primarily on physical (e.g. heat) methods of drying oily sludge.

Strengths: This manuscript provides a novel method of speeding up the drying processes of industrial oily sludge. The authors use an orthogonal experimental design and an efficient set of experiments to determine the most appropriate amendments to facilitate drying. Through this work they are able to decrease the semi-drying time of oil sludge by 2/3. Generally the manuscript is well written and has been edited.

Weakness: While generally a solid study on a novel method of handling oily sludge authors overstate the contribution of their work to our mechanistic understanding of the drying processes. Additionally, the study lacks statistical rigor requiring cautious interpretation of results.

Given the overall value of the research conducted, I recommend this manuscript for publication once appropriate revisions have addressed the concerns and editorial suggestions elaborated below.

Specific Areas for Improvement

Major

While generally a solid study and well written, there are several areas that I authors should address. First, the manuscript overstates its contribution to the mechanistic process of drying (e.g. lines 217 – 235, 258-270, 317-335, 343-371). While molecular-level mechanisms of drying discussed in the text are supported by the data presented, I do not think the data from this study provides direct evidence of these mechanisms. I suggest that reframing the discussion of mechanisms as possible explanations,

consistent with observed data would be appropriate and more accurate as the mechanisms themselves are not explored in detail by the research performed. This should also involve the addition of relevant references to these sections of the text.

Second, a lack of statistical analysis of the data is also noted. The authors provide no information on sample replicates performed or calculation of errors, yet throughout the manuscript observed changes are noted as either significant or not significant (e.g. lines 202, 209, 239). If multiple experiments were carried out and errors on measurements are known, these values must be reported, especially if, as I suspect, variability in moisture content is high among different samples. Percent moisture values measured by the authors span less than 10% variability across these 11 treatments (and blank). Having some idea of the variability on these measurements would make it easier to accept the results of this study and allow greater understanding of the drying processes. While replicates and thorough statistical analyses are not always common practice in the field at this point, analysis of at least one sample in triplicate to provide some idea of variability would be extremely valuable. If available, error bars should be added to figures to aid the reader in accurate interpretation of the data. If authors do not have error data for their experiments and are not able to obtain this data, language of the manuscript must be revised and it should be clearly stated that each experiment was only performed once.

Minor

Third, authors should address the application of these experiments to larger-scale industrial processes that they discuss during the introduction. The experiments were carried out on very small sample sizes (10 mg), how do they anticipate their result scaling up? Is this research relevant to larger scale drying where surface area/volume ratios will be dramatically different? What recommendations do authors have for scaling for industrial application? What problems do they foresee?

Fourth, authors should briefly describe why modified materials were selected. I suggest elaborating on the discussion of the various OSM materials used (lines 300-310), adding appropriate references and moving it forward in the manuscript (to the introduction/Methods: Experiment section). It would be useful for the reader to understand why the various amendments chosen would be expected to aid in the drying processes prior to the OSM selection and experimental results.

Finally, authors must clean up language and clarify to reader the different forms of water discussed in the manuscript. This should include a clear description of each term used and consistent use of this terminology throughout the manuscript. There are currently over ten different terms used throughout the manuscript. (currently in use: free water, gap water, surface water, combined water, internal free water, pore water, absorbed water, combined water, water in emulsion, interstitial water, free emulsion…)

While the English of the manuscript is generally good there are still numerous typographical errors and areas that could use clarification. In addition to addressing these errors I encourage authors to complete a thorough read through of the paper to ensure their intended meanings have not been changed through the editing process. While not complete, below is a list of some specific suggestions and questions I noted as I read through the manuscript.

Notes on Figures and Tables

General: (1) Figure captions and Table headings could be more informative.

(2) Formatting for all tables could be improved. Values should be vertically centered and headings bolded.

Figure 3: Suggest authors graph this figure as % moisture vs Time. Current format is difficult to read and is inconsistent with how figure is discussed in the text. (Note: units on x-axis are currently incorrect)

Figure 6: Figure does not add to text or aid in data interpretation. Delete?

Figure 7: Interesting figure, currently not labeled sequentially (current: a, b, d, c …) which is confusing. Units for weight reduction per second should be listed. Y-axis (moisture content of oily sludge sample) is mislabeled.

Figure 8: define TGA in caption.

Figure 9: Suggest flipping this figure so transition process is shown below general start/end schematics to help reader. There are no clear structural changes between the initial and final schematics while text (lines 399 – 401) discusses a change in porosity. Suggest adjusting schematic to illustrate this change in porosity. Unclear where in the figure "free water" is shown. "thin holes" in final schematic discussed as "channels" in the text.

Table 1: remove "number" column unless used elsewhere.

Tables 2 and 3. Unless germane to the analysis/outcome I would suggest authors reorder tables so that they read in order A, B, C, and D as discussed in the text.

Table 3: no explanation or discussion of bottom portion of table (R/K values). If unnecessary should be removed. If kept should be discussed in text.

Line-by-Line notes:

- Line 25: "OSM increased…" increased how? by mass? Volume?
- Line 43: add "e.g." to description – not all oily sludge has the same oil/water content, numbers provided are only one example of the composition of an oily sludge.
- Line 52: move/edit "These approaches also consume significant energy." to line 54, before sentence on solar dryers.
- Line 57: delete "continuously" (not necessary)
- Line 57: "the analysis above" – There is no analysis above, and no discussion of natural wind drying. Statement is misleading. How wind better than solar? wind also varies.
- Line 71: "this leads to the need" … - no it does not, but it would be desirable to have a shorter drying period there is no evidence provided for a specific need of a drying time in fewer than 5 days.
- Line 76: edit – "wide source" → "widely available source"
- Line 82: need references – what studies?

- Line 114: “m,g” very confusing. I believe g is the units, unsure of m, mass? clarify
- Line 117: is it true that the material with the lowest moisture content was selected? Text states moisture content varied from 68-72% (line 102), table 1 says 71%
- Line 120: “Distilled water was used…” as a reference material?
- Lines 122-137: Wording could be simplified to facilitate understanding. A labeled schematic identifying each "m" would be useful for deciphering what each of these masses refers to. Supplemental figure?
- Line 137: Equation 4 does not provide a rate as is stated in the text.
- Line 140: define TGA
- Line 146: unclear: 100-500 mg is this the mass of the water or the sludge sample?
- Line 150: define SEM
- Line 154: SEM experiments = images collected?
- Line 155: use correct symbol for micro
- Line 170: use more appropriate word
- Line 175: “component of OSM” → “combination of OSM components”?
- Line 195: unclear as to if this formulation was used for all remaining experiments. If so add a sentence stating as much.
- Line 197: delete “adding”
- Line 198: delete “Effect of OSM Addition.”
- Lines 201-213: Authors suggest significant differences between experimental treatments but do not provide statistics to back up this claim.
- Line 208: There was an upward trend in what?
- Line 209-210: Data not shown – clarify for reader that this data is not presented
- Line 210-211: Moisture content of what was higher compared to blank?
- Line 215: edit - “dropped below 60% for all OSM treatments”
- Line 215-216: How do the curves indicate to authors which processes are responsible? This statement appears to be a reference to Zhu et al. I am not sure authors intend to reference the actual shape of their curves. How are points 1 and 2 visually seen in these curves. This is an example of where authors appear to discuss explanations of data as a direct product of the research performed. Strongly suggest authors take a close look at this section and edit for clarity.
- Line 234: moisture content dropped below 60% for the OSM amended treatments only. Clarify.
- Line 250-251: Confusing. Edit for clarity
- Lines 254-256: Confusing. Edit for clarity.
- Line 255: edit – “should be controlled at no less than…” →”should be controlled to within at least…”. Unclear where this conclusion is coming from.
- Line 262: past tense: “evaporation area was…”
- Line 264: Surface concentration of what?
- Section starting at 257: Generally section should be copy-edited for clarity. This study provides data in support of mechanisms but does not specifically determine the mechanisms itself. Additionally numerous unsubstantiated uses of “significant”. See weaknesses discussed above.
- Lines 268-269: These two sentences seem to directly contradict eachother. I am unclear as to the authors intent. For the emulsion or for the water? Rewrite for clarity
- Line 284 – 289: How is this data shown in the images?

- Line 296:"hydrophilicity increased and the hydrophilicity decreased"
- Line 297-298: Confusing. Edit for clarity.
- Section starting at line 300: Add references. Suggest relocating to Experiment or Introduction section.
- Line 312: raw data should be removed.
- Line 327/331/333: Continue guiding reader. ".. reduction was gradually reduced (c to e phase)." etc
- Line 338: "stabilizing after ~1000 s"
- Line 343-371: Confusing / feels redundant with previous section. One discusses data where no OSM was added the other with OSM addition – distinction is not clear in text and adds to confusion. Addition of the "four stages" of the drying process to the relevant figure (figure 8?) would be extremely helpful. Additionally, use of multiple terms to describe water forms adds to confusion. Simplify and be consistent
- Line 391: Delete "f"
- Line 395: edit – "This dense …closed space" → "Forming a dense surface structure with limited routes for water migration"?
- Line 397: "oily sludge amendments increased"?
- Line 408: I believe authors used an OSM mixture, not compound for their experiments (as noted above this should be clarified in the methods section)
- Line 410: missing period. "…disposal cost. The environmental …"
- Line 411: Again, unsure where this conclusion has come from, figure 3? Lines 254-255. Clarify.
- Line 475/486: references 18 and 22 are missing year information
- References: generally inconsistent formatting of dates across references
