# Supplementary material for: Facilitating the natural semi-drying of oily sludge by changing the form of water
Source: PLoS One. 2021 Jan 14;16(1):e0245430. doi: 10.1371/journal.pone.0245430 (PMC7808663; doi:10.1371/journal.pone.0245430)
Supplement: Attachment — Submitted filename: Manuscript ReReview - Liu (2020).pdf [file pone.0245430.s004.pdf]

Manuscript Review: Promoting the natural semi-drying of oily sludge by changing the form of water (Liu et al., 2020)

Authors have toned down the language surrounding their presentation of the mechanistic description of the drying process. I believe authors have adequately addressed this concern overall.

Authors state they have run the experiment again (in triplicate?) allowing errors bars to be added to several figures making data interpretation more reliable. However, these error bars and how they were calculated (e.g. based on triplicate analysis of one sample or based on running the experiment in triplicate) have not been discussed in the text. The meaning and derivation of these error bars needs to be added to the text and figure captions. With addition of supporting text I believe authors have adequately addressed this concern.

Authors have expanded and moved forward a discussion of the various OSM materials. Currently, the primary issue I have regarding these materials is the complexity of Table 3 in particular as well as the unclear discussion regarding the actual materials used in the OSM mixture chosen. Authors state an initial screening of each reagent was performed. Language is missing / confusing regarding the initial screening carried out by the authors. From my reading each potential reagent (multiple from each type; listed in table 1) was analyzed for impact on % moisture (results listed in Table 1). From that initial experiment only 1 reagent from each category was chosen for the final OSM mixture (Lines 123-124). This would indicate that the remaining experiments performed based on their orthogonal experiment results use an OSM mixture that contains only 4 reagents, 1 from each different category. This is not stated in the manuscript. It should be clearly stated what the selected OSM formulation was composed of for their experiments. Lines 206-208 attempt to do this but only list the overall types and imply that all reagents listed in table 1 were kept for their final OSM mixture that was tested.

Authors have notably cleaned up language regarding the different types of water. However, there are still at least 7 terms I counted that are in use. I suspect these could be further simplified. (currently in use: free water, combined water, bound water, separated water, gap water, surface water, surface-bound water…)

There remain numerous typographical errors and areas that require clarification. Most notably the Table 3 description is still inadequate. I have done my best to provide detailed comments and suggested edits to aid in simplifying language, correcting grammar, and clarifying the author's intent to help improve overall readability. I encourage authors to complete a thorough read through of their manuscript to ensure their intended meanings have not been changed through the editing process. I also encourage authors to seek editing from someone outside of the experiment who can provide an outsiders perspective on the clarity of their descriptions. While not complete, below is a list of some specific suggestions and questions I noted as I read through the manuscript.

<u>Notes on Figures and Tables</u>

Significant improvements have been made to several figures and the authors efforts here are noted. It also appears authors have moved quite a few figures into supporting information?? Several figures are now labeled with an "S" prefix (now labeled as Figure S2 etc). Several of these figures are germane to the initial discussions of the authors and these figures present most of the data for their study so I am a bit unclear as to the motivation for this decision. Or perhaps I am misinterpreting something? Regardless, while the efforts at improving figures are notable some additional edits/suggestions/confusions still remain.

General:

(1) Figure captions and Table headings should be more informative. Tables and figures should stand along and be understandable as to what data they are presenting without the need for the text. Figure caption and Table heading should be expanded.

(2) Table headings should be bolded and formatting consistent across tables (e.g. Tables 1 and 3)

Figure 2: Numerous typos, and erronious capitalizations in the figure caption.

Figure 3: I appreciate the text added by authors to the text discussing the surface tension results. I still do not believe this figure aids in the data interpretation or discussion. I especially don't see why this is a figure that has been kept in the main text while several more informative figures have been seemingly moved to the SI.

Figure 5: define TGA, read for typos and expand caption.

Table 1: Suggest changing first column heading to either "additive type" or "OSM type"

Table 3: I appreciate the authors efforts to clarify the bottom half of Table 3 in the manuscript text, however, with the description provided I am still unable to determine where these values are coming from (particularly the Ki values). I also believe that Table 3 needs to be two tables. The bottom half of this table introduces a new column (moisture content after 5 days). This indicates that this data should be separated and discussed in a separate table. I also encourage authors to expand on table headings for both the top part of Table 3 and the new table formed to describe to the reader the data being presented.

Units: For ease of reading I suggest authors use a consistent and format for presenting units. Currently all figures present units in parenthesis (e.g. time (h)). This is an appropriate format that is easily to understand. I encourage authors to review their manuscript text (e.g. lines 120 and 143) and table headings (e.g. Tables 1 and 3) to make this formatting consistent across the manuscript.

<u>Line-by-Line notes:</u>

- Lines 26-17: semi-colons should be commas
- Line 48: missing space before "Free" and unrelated sentence at end that needs to be deleted

- Line 54: reduce moisture content to below 50%? Having a value here would be informative
- Line 55: and natural drying? Discussed later in paragraph.
- Line 58: → "…the high cost of these approaches…"
- Line 62: "given that" → "as"
- Line 63-65: Move "similar to solar drying." "…natural air drying, similar to solar drying, is the most economical …to a semi-dry state."
- Line 72/80: Natural drying vs natural wind drying are you making a distinction here? Delete "wind" or clarify distinction…can add air movement as a factor.
- Line 74: add sentence and number reasons that follow: "there are several reasons a shorter drying period would be advantageous 1) operating…2) longer storage times…"
- Line 78: delete "and then transport it to the brick factory"
- Line 81: "…Putrescible smell in…" → "…putrid smell of…"
- Line 89: delete yet
- Line 90: if this accurately describes your work I suggest "In this study we examine how the form of water in an oily sludge can be modified using a chemical agent to speed up the drying process …"
- Line 105-107: move figure 1 reference to end of second sentence.
- Line 112: separated water = free water?
- Line 116: reduce → decrease
- Line 123: the material with the lowest moisture content for each additive type? (match language in table 1, column 1). My understanding is this led to 1 material from each additive type being chosen for the tested OSM mixture using in the orthogonal experiments. If this is the case this should be specified and the individual OSM additives indicated. If this is not the case it should be clarified what is meant by the selection resulting from this initial screening.
- Line 126-127: rewrite/combining of last two sentences: "Surface tension (…) of the OSM was measured at room temperature with DI as a reference material."
- Line 129: suggested rewrite for simplicity: "…assessed by weighing an oil sludge sample (m_0) and then heating to a constant weight (m_0')."
- Line 137-139: Suggested edit for clarity: "The sample was then dried at 80C until it reached a constant mass (tfinal). Weight percent moisture for samples at t1 and t2 and the moisture content reduction rate were calculated according to equations 2-4."
- Line 142: modify equation so that equation provides a rate: $R = \frac{W_{t1}\% - W_{t2}\%}{|t_1 - t_2|}$
- Line 143-146: Wording has been modified but is still rather confusing. Suggest changing to "where m1, m2, m3, m4, m5 and m6 are masses of the beaker, added oily sludge, added OSM or chemical agent, and masses recorded at t1 and t2 and tfinal respectively. R is the moisture content reduction rate.
- Line 148: "…placed in the sample chamber…"
- Line 149: remove all caps of Mettler Toledo
- Line 149: delete: "a controlled temperature of" unnecessary
- Line 152: suggested edit "(1) oil sludge (100-500 mg) was placed on the slide and gently covered with a glass slide to observe the …"
- Line 153-154: suggest replacing "the oily sludge sample with the added OSM was then placed on a slide, gently covered with a glass slide, and observed for the presence of water under the microscope"

with “this was compared with the oil sludge sample with the added OSM which was similarly examined” to simplify.

- Line 156: “was” → “were”
- Line 157: the oily sludge leachate with the OSM was placed on the slide? Or just the oily sludge leachate?
- Line 156-158: how did this help identify the different forms of water?
- Line 160: SEM “experiments were conducted” = “images were collected”? 10 and 2 um = resolution?
- Line 175: “…compounding effects.”
- Line 177: “assess the four types of chemical agents listed in Table 1” (language should match column heading in Table 1)
- Line 179: Inogranic gelling materials = inorganic cementous materials? If yes, suggest replacing to avoid introduction of new term.
- Line 179: add “and”: “and other regents and were labeled A…”
- Line 197-202: I appreciate efforts made by authors to explain the K values but I do not find this description adequate. I am unclear where the Ki values come from in particular. I appreciate explanation of R and ki value significance and final result (A1B2C3D3) is understood but description of K values is inadequate. Also, as mention earlier, unclear if A, B, C and D are only one of each of the chemical agents listed in table 1 or if all chemical agents of a certain group were used in this analysis. This must be clarified
- Line 207-208: missing spaces after % symbols…clarify if all emuslifiying/oxidants etc were used or only 1 (see previous comments).
- Line 210+: Discussion of results should be in the present tense, not past tense. For example: “data are shown in Figure S1”. Not “data were showed in Figure S1”.
- Line 211/212: Suggested sentence rewrite: “Changes in the moisture content over time are shown in Fig. S1.”
- Line 214/215: “…the lower the final moisture content of the oily sludge at the end of the experiment.”
- Line 215: “the effect of OSM” → “OSM content”
- Line 216: Clarification, the OSM was added at the beginning of the experiment, not after 24 hours correct? If this is the case suggest modifiying “after adding the OSM” to “with the addition of the OSM”. Similarly, see comment for line 220.
- Line 220: “After the OSM was added 24 h” → “Twenty-four hours after the OSM was added the moisture content…” see comment for line 223.
- Line 223: “when it was added at the beginning” → “when it was initially added”. Currently reads as though there were two experiments, one where the OSM was added at t0 and another where the OSM was added after 24 hours. I believe this is not what you intend/did.
- Line 229: “At this time point, moisture content dropped below…”
- Line 229-232: rewrite: “The curves of moisture content reduction rate in Fig. S1 show two trends: fast moisture content reduction during the first 24 hours and rates slowly decreasing after 24 hours. These trends are consistent with the processes of evaporation and diffusion as described by Zhu et al., 2012”
- Line 237: add somewhere in this paragraph how this trend is observed in the data. For example “This is seen in the initially high moisture content reduction rate” … add similar description for diffusion paragraph below.

- Line 251: would you accurately say the initial low to high moisture content reduction was due to evaporation and the gradual decrease after 24h due to diffusion?
- Line 258: increase in the mass fraction of what?
- Line 264: “was smaller” → “became slower”?
- Line 265: “evaporation surface area.”
- Line 266-267: not a sentence. Confusing, edit for clarity.
- Line 268: Raising → Increasing
- Line 270: “required semi-dried” → “required to be semi-dry”
- Line 271: “temperature should be maintaining at greater than 10°C”
- Line 271: Figure S2??
- Section 274: leachate or emulsion. Choose one.
- Line 286: over time?
- Line 291: were coating thickness and area of emulsion surface measured?
- Line 297-300: great text for figure 2 caption
- Line 302: Figure 2b??
- Line 303: leaching solution → leachate
- Line 304: situation → observations
- Line 316: what are double layers?
- Line 320: what is “It”? The surfactant?
- Line 321: electricity → negatively charged species??
- Line 324: define DSC
- Line 325: “…was reduced by 69% after drying for 10 min at 100°C (Fig. 4).”
- Line 344: missing “free water”? and combining of sentences. “…the interior free water of the oil sludge quickly increased resulting in increased evaporation (e to f).”
- Line 330-331: delete “(a,b,c,d,e,…for the evaporation rate)” and place in caption (already there).
- Line 331-332: “the rate was reduced between b and c as remaining free water is evaporated”
- Line 333-334:”oily sludge was demulsified and the free water increased over a short time leading to an increase in the rate of mass loss (c to d phase).
- Line 334-336: “As the emulsified water decreased, the heat was …to the interior, and the bound water…”
- Line 336: “continuously reduced” → “decreased”
- Line 347: emulsion evaporated? New sentence: Most of the …? Rewrite.
- Line 350: “were shown in” → “are shown in”
- Line 352: delete significantly
- Line 353: edit – “…for the non-amended oily sludge…”
- Line 355: “four stages to the drying processes as outlined below.” These four stages are not clearly shown in figure 5. Describe how these are shown in the figure.
- Line 359-360: confusing. Edit for clarity.
- Line 396: unbold From
- Line 398: delete “the”
- Line 414: best effect = most important effect? – how was this determined?
- Line 421-425: accidental sentence? Reread and edit.

- Line 425-428: “At environmental temperatures exceeding 10°C the OSM significantly shortened the natural semi-drying time of the oily sludge from ___ to __”
- Line 432: facilitating
- Line 433: speeding up
