# Supplementary material for: Facilitating the natural semi-drying of oily sludge by changing the form of water
Source: PLoS One. 2021 Jan 14;16(1):e0245430. doi: 10.1371/journal.pone.0245430 (PMC7808663; doi:10.1371/journal.pone.0245430)
Supplement: Attachment — Submitted filename: Response to reviewers.docx [file pone.0245430.s005.docx]

**Dear Editors and Reviewers,**

Thank you for your letter and for the reviewers’ comments concerning our manuscript entitled “**Facilitating the natural semi-drying of oily sludge by changing the form of water**” (PONE-D-20-21667R1). Those comments are all valuable and very helpful for revising and improving our paper. We have studied comments carefully and have made correction which we hope to meet with approval. Revised portion are marked in red or blue in the paper. The main corrections in the paper and the responds to the reviewers’ comments are as following.

Yucheng Liu

On behalf of all co-authors

Reviewer #1:

Authors state they have run the experiment again (in triplicate?) allowing errors bars to be added to several figures making data interpretation more reliable. However, these error bars and how they were calculated (e.g. based on triplicate analysis of one sample or based on running the experiment in triplicate) have not been discussed in the text. The meaning and derivation of these error bars needs to be added to the text and figure captions. With addition of supporting text I believe authors have adequately addressed this concern.

**Responds to the reviewers’ comments:**

Thanks for your comments. Your suggestion is greatly appreciated.Yes, based on running the experiment in triplicate  of one sample,each experimental data shown in the figure is based on the average of three parallel experiments, the specific data of the three experiments is in the supporting information. The calculation principle of error bars is to use the standard error of the mean(note as SE) to reflect the degree of dispersion of data. Standard deviation(note as SD) describes the degree of clustering of data around the mean.$SE=\frac{\mathrm{SD}}{\sqrt{N}}$, N is the number of data points,$\mathrm{SD}=\sqrt{\frac{\sum\left( X_{i}-M \right)}{N}}$, M is the value of a single data point, M is the mean. Error bars is -1SE to +1SE. The meaning and derivation of these error bars had been added to the text.(Line 123:‶ Each experimental data in this article takes the average of three parallel experiments, and uses the standard error of the mean to reflect their degree of dispersion.″)

Reviewer #2:

Authors have expanded and moved forward a discussion of the various OSM materials. Currently, the primary issue I have regarding these materials is the complexity of Table 3 in particular as well as the unclear discussion regarding the actual materials used in the OSM mixture chosen. Authors state an initial screening of each reagent was performed. Language is missing / confusing regarding the initial screening carried out by the authors. From my reading each potential reagent (multiple from each type; listed in table 1) was analyzed for impact on % moisture (results listed in Table 1). From that initial experiment only 1 reagent from each category was chosen for the final OSM mixture (Lines 123-124). This would indicate that the remaining experiments performed based on their orthogonal experiment results use an OSM mixture that contains only 4 reagents, 1 from each different category. This is not stated in the manuscript. It should be clearly stated what the selected OSM formulation was composed of for their experiments. Lines 206-208 attempt to do this but only list the overall types and imply that all reagents listed in table 1 were kept for their final OSM mixture that was tested.

**Responds to the reviewers’ comments:**

Thanks for your comments. Your suggestion is greatly appreciated. The data in Table 3 has been split into 2 tables (Table 4 has been added), the description of the K value calculation method in Table 4 has been re-added, and the specific groups of the 4 additive types selected in Table 1 have been clearly stated Minute(Aminosulfonic acid, Fe_2_(SO_4_)_3_, Portland cement ，MgCl_2_,). Added in the text, ‶ Aminosulfonic acid, Fe2(SO4)3, Portland cement ，MgCl2, they were only one of each of the chemical agents listed in Table 1.″

Reviewer #3:

Authors have notably cleaned up language regarding the different types of water. However, there are still at least 7 terms I counted that are in use. I suspect these could be further simplified. (currently in use: free water, combined water, bound water, separated water, gap water, surface water, surface- bound water…)

**Responds to the reviewers’ comments:**

Thanks for your comments. Your suggestion is greatly appreciated. The water in oily sludge mainly exists in three forms: free water, bound water, and emulsified water. Free water refers to water that can flow freely, and bound water refers to the attraction of electrically charged molecules to the internal pore surfaces of oily sludge particles. Water on the external surface, emulsified water refers to oil-water emulsion, which is a water-in-oil, oil-in-water or multiple emulsified dispersion system.( End of the first paragraph)

This guidance had been very helpful to me, the full text has been revised, the main forms of water for free water, bound water, emulsified water, combined water is modified to bound water, separated water and gap water is modified to free water, surface water, surface- bound water is modified to (Particle surface) bound water. Here are some changes about different forms of water, more details please see the manuscript marked in red.

Reviewer #4:

There remain numerous typographical errors and areas that require clarification. Most notably the Table 3 description is still inadequate. I have done my best to provide detailed comments and suggested edits to aid in simplifying language, correcting grammar, and clarifying the author’s intent to help improve overall readability. I encourage authors to complete a thorough read through of their manuscript to ensure their intended meanings have not been changed through the editing process. I also encourage authors to seek editing from someone outside of the experiment who can provide an outsiders perspective on the clarity of their descriptions. While not complete, below is a list of some specific suggestions and questions I noted as I read through the manuscript.

**Responds to the reviewers’ comments:**

Thanks for your comments. Your suggestion is greatly appreciated. The data in Table 3 has been split into 2 tables (Table 4 has been added), the description of the K value calculation method in Table 4 has been re-added. According to your suggestion, we have sought out 2-3 people outside the experiment and carefully checked this paper from their academic perspective to help the paper description more clearly. For example:

Line 204:Added‶ The values in Table 4 are calculated from the experimental results in Table 3.″

Full text native language editing.e.g Certification, A highlighted Blue Sentence in Revised Manuscript with Track Changes.

Reviewer #5:

Significant improvements have been made to several figures and the authors efforts here are noted. It also appears authors have moved quite a few figures into supporting information?? Several figures are now labeled with an “S” prefix (now labeled as Figure S2 etc). Several of these figures are germane to the initial discussions of the authors and these figures present most of the data for their study so I am a bit unclear as to the motivation for this decision. Or perhaps I am misinterpreting something? Regardless, while the efforts at improving figures are notable some additional edits/suggestions/confusions still remain.

**Responds to the reviewers’ comments:**

Thank you very much for your suggestions, which will greatly improve the readability of our paper. We have put the main data graphs in Manuscript, and the corresponding experimental data sheets are used as supporting materials in Supporting Information. In addition, their titles have been corrected accordingly.

Fig. S1, Fig. S2, Fig. S3, had been modified to Fig. 2(Table S1- able S2), Fig. 3(Table S3), Fig. 4(Table S4- able S5).

Fig. 3 had been modified to Fig. S1, And added to the supporting information.

Reviewer #6:

(1) Figure captions and Table headings should be more informative. Tables and figures should stand along and be understandable as to what data they are presenting without the need for the text. Figure caption and Table heading should be expanded.

**Responds to the reviewers’ comments:**

Thanks for your comments. Your suggestion is greatly appreciated. We have modified it（e.g. Fig.2 and Table 4）.

(2) Table headings should be bolded and formatting consistent across tables (e.g. Tables 1 and 3)

**Responds to the reviewers’ comments:**

Thanks for your comments. Your suggestion is greatly appreciated. Table headings had been bolded and formatting consistent across tables (e.g. Tables 1 and 3，Fig 5)

Reply:

Thank you for your very detailed suggestions. I have checked and revised them item by item. The details are as follows:

| suggestions and questions | Revision/description |
| --- | --- |
| Figures | |
| Figure 2: Numerous typos, and erronious capitalizations in the figure caption. | Fig.5: We have redrawn the figure: Great text line 297-300 for figure 5 caption |
| Figure 3: I appreciate the text added by authors to the text discussing the surface tension results. I still do not believe this figure aids in the data interpretation or discussion. I especially don’t see why this is a figure that has been kept in the main text while several more informative figures have been seemingly moved to the SI. | We have redrawn the figure ,Fig.3: Fig. 3 had been modified to Fig. S1, And added to the supporting information. |
| Figure 7: define TGA, read for typos and expand caption. | Figure 7: We have modified it.“**Thermo gravimetric analysis (TGA) curvs**” |
| Table | |
| Table 1: Suggest changing first column heading to either “additive type” or “OSM type” | We have modified it. |
| Table 3: I appreciate the authors efforts to clarify the bottom half of Table 3 in the manuscript text, however, with the description provided I am still unable to determine where these values are coming from (particularly the Ki values). I also believe that Table 3 needs to be two tables. The bottom half of this table introduces a new column (moisture content after 5 days). This indicates that this data should be separated and discussed in a separate table. I also encourage authors to expand on table headings for both the top part of Table 3 and the new table formed to describe to the reader the data being presented. | We have redrawn the table, Split Table 3 into two tables,and added new table heading for table 4:‶ **Data processing results table**″ , for example, when the level number of K_1_ is 1, and the corresponding level number of factor A is 1, K_1_ is equal to the sum of the results of the moisture content measurement data in each row when A is 1 in Table 3, |
| Line-by-Line notes: | |
| Lines 26-17: semi-colons should be commas | Lines 27-28:We have modified it. |
| Line 48: missing space before “Free” and unrelated sentence at end that needs to be deleted | Lines 47-48: We have modified it. Added space before “Free” and unrelated sentence at end that had been deleted. |
| Line 54: reduce moisture content to below 50%? Having a value here would be informative | Line 53: We have modified it. reduce moisture content to below 50%. |
| Line 55: and natural drying? Discussed later in paragraph. | Line 53: The oily sludge used to produce bricks requires drying to reduce moisture content to below 50%，and drying methods Discussed later in Line57-83, includes natural drying |
| Line 58:  “…the high cost of these approaches…” | Line58: We have modified it. |
| Line 62: “given that”  “as” | Line 61: We have modified it. |
| Line 63-65: Move “similar to solar drying.” “…natural air drying, similar to solar drying, is the most  economical …to a semi-dry state.” | Lines 62-63: We have modified it. |
| Line 72/80: Natural drying vs natural wind drying are you making a distinction here? Delete “wind” or  clarify distinction…can add air movement as a factor. | Lines 70/78: We have modified it. Line 75 clarify distinction…can add air movement as a factor. Line 87, We have Deleted “wind”. |
| Line 74: add sentence and number reasons that follow: “there are several reasons a shorter drying  period would be advantageous 1) operating…2) longer storage times…” | Lines 73-75: We have modified it. |
| Line 78: delete “and then transport it to the brick factory” | Line77: We have modified it. |
| Line 81: “…Putrescible smell in…”  “…putrid smell of…” | Line80: We have modified it. |
| Line 89: delete yet | Line88: We have modified it. |
| Line 90: if this accurately describes your work I suggest “In this study we examine how the form of water in an oily sludge can be modified using a chemical agent to speed up the drying process …” | Lines 88-90: We have modified it. “In this study we examine how the form of water in an oily sludge can be modified using a chemical agent as a modified material (OSM) to speed up the drying process.” |
| Line 105-107: move figure 1 reference to end of second sentence. | Lines 105-107: We have modified it. |
| Line 112: separated water = free water? | Line 112: We have modified separated water to free water |
| Line 116: reduce to decrease | Line 115: We have modified it. |
| Line 123: the material with the lowest moisture content for each additive type? (match language in table 1, column 1). My understanding is this led to 1 material from each additive type being chosen for  the tested OSM mixture using in the orthogonal experiments. If this is the case this should be specified and the individual OSM additives indicated. If this is not the case it should be clarified what is meant by the selection resulting from this initial screening. | Lines 118-123: We have modified it. Added a screening method “For each of the above 4 additive types of materials, the single agent was tested and compared with the blank sample. The materials with water content higher than the blank sample were deleted, and choose the material with the lowest moisture content for each additive type for the tested OSM mixture using in the orthogonal experiments. ” |
| Line 126-127: rewrite/combining of last two sentences: “Surface tension (…) of the OSM was measured at room temperature with DI as a reference material.” | Line130-131: We have modified it. |
| Line 129: suggested rewrite for simplicity: “…assessed by weighing an oil sludge sample (m 0 ) and then  heating to a constant weight (m 0 ’).” | Line133: We have modified it. |
| Line 137-139: Suggested edit for clarity: “The sample was then dried at 80C until it reached a constant mass (tfinal). Weight percent moisture for samples at t1 and t2 and the moisture content reduction rate were calculated according to equations 2-4.” | Line 141-144: We have modified it. |
| Line 142: modify equation so that equation provides a rate: $R=\frac{W_{t_{1}}\%-W_{t_{2}}\%}{\left\vert t_{1}-t_{2} \right\vert}$ | Line147: We have modified it. |
| Line 143-146: Wording has been modified but is still rather confusing. Suggest changing to “where m1,  m2, m3, m4, m5 and m6 are masses of the beaker, added oily sludge, added OSM or chemical agent,  and masses recorded at t1 and t2 and tfinal respectively. R is the moisture content reduction rate. | Line148-150: We have modified it. |
| Line 148: “…placed in the sample chamber…” | Lines 153: We have modified it. |
| Line 149: remove all caps of Mettler Toledo | Line 154: We have modified it. |
| Line 149: delete: “a controlled temperature of” unnecessary | Line154: We have modified it. |
| Line 152: suggested edit “(1) oil sludge (100-500 mg) was placed on the slide and gently covered with a glass slide to observe the …” | Line 157: We have modified it. |
| Line 153-154: suggest replacing “the oily sludge sample with the added OSM was then placed on a slide, gently covered with a glass slide, and observed for the presence of water under the microscope”  with “this was compared with the oil sludge sample with the added OSM which was similarly examined” to simplify. | Line 157-158: We have modified it. “this was compared with the oil sludge sample with the added OSM which was similarly examined” |
| Line 156: “was”  “were” | Line 160: We have modified it. |
| Line 157: the oily sludge leachate with the OSM was placed on the slide? Or just the oily sludge leachate? | Line 161: We have modified it. |
| Line 156-158: how did this help identify the different forms of water? | Line 162-163: We have modified it. Added ”Under the microscope, the emulsified water appears as an opaque droplet with outer ring, while the free water is relatively uniform and transparent.  ” |
| Line 160: SEM “experiments were conducted” = “images were collected”? 10 and 2 um = resolution? | Line 165-169：We have modified it. “he samples were dried to a constant weight at 80 °C, a small amount of sample powder was attached to the copper platform, and then the sample is sprayed with gold to increase the conductivity of the sample, so that the image was clear and stable. The gold sprayed samples were placed in a vacuum system in the Scanning electron microscope, vacuumed and scanned. During the test, images were displayed by a computer system, and the results were recorded at 10 μm and 2 μm resolutions, respectively.” |
| Line 175: “…compounding effects.” | Line 184: We have modified it. |
| Line 177: “assess the four types of chemical agents listed in Table 1” (language should match column  heading in Table 1) | Line 186: We have modified it. |
| Line 179: Inogranic gelling materials = inorganic cementous materials? If yes, suggest replacing to avoid introduction of new term. | Line 188: We have modified it. choose“inorganic cementous materials” |
| Line 179: add “and”: “and other regents and were labeled A…” | Line 188: We have modified it. |
| Line 197-202: I appreciate efforts made by authors to explain the K values but I do not find this description adequate. I am unclear where the Ki values come from in particular. I appreciate explanation of R and ki value significance and final result (A1B2C3D3) is understood but description of K values is inadequate. Also, as mention earlier, unclear if A, B, C and D are only one of each of the chemical agents listed in table 1 or if all chemical agents of a certain group were used in this analysis.  This must be clarified | Line 205-210: for example, when the level number of K_1_ is 1, and the corresponding level number of factor A is 1, K_1_ is equal to the sum of the results of the moisture content measurement data in each row when A is 1 in Table 3. Aminosulfonic acid(marked as A), Fe_2_(SO_4_)_3_(marked as B), Portland cement(marked as C), MgCl_2_(marked as D) |
| Line 207-208: missing spaces after % symbols…clarify if all emuslifiying/oxidants etc were used or only 1 (see previous comments). | Line 218-219: We have modified it. Added“A, B, C and D are only one of each of the chemical agents listed in table 1”. Aminosulfonic acid(marked as A), Fe_2_(SO_4_)_3_(marked as B), Portland cement(marked as C), MgCl_2_(marked as D) |
| Line 210+: Discussion of results should be in the present tense, not past tense. For example: “data are shown in Figure S1”. Not “data were showed in Figure S1”. | Line 223: We have modified it. |
| Line 211/212: Suggested sentence rewrite: “Changes in the moisture content over time are shown in Fig. S1.” | Line 222-223:We have modified it. |
| Line 214/215: “…the lower the final moisture content of the oily sludge at the end of the experiment.”  - Line 215: “the effect of OSM”  “OSM content” | Line 225-226:We have modified it. |
| Line 216: Clarification, the OSM was added at the beginning of the experiment, not after 24 hours  correct? If this is the case suggest modifiying “after adding the OSM” to “with the addition of the  OSM”. Similarly, see comment for line 220. | Line 227/231:We have deleted it, |
| Line 220: “After the OSM was added 24 h”  “Twenty-four hours after the OSM was added the moisture content…” see comment for line 223. | Line231-232: We have modified it. |
| Line 223: “when it was added at the beginning”  “when it was initially added”. Currently reads as  though there were two experiments, one where the OSM was added at t0 and another where the OSM  was added after 24 hours. I believe this is not what you intend/did. | Line 234: We have modified it. |
| Line 229: “At this time point, moisture content dropped below…” | Line 240:We have modified it. |
| Line 229-232: rewrite: “The curves of moisture content reduction rate in Fig. S1 show two trends: fast moisture content reduction during the first 24 hours and rates slowly decreasing after 24 hours. These trends are consistent with the processes of evaporation and diffusion as described by Zhu et al., 2012” | Line 240-244:We have modified it. |
| Line 237: add somewhere in this paragraph how this trend is observed in the data. For example “This is seen in the initially high moisture content reduction rate” … add similar description for diffusion paragraph below. | Line 247/253-254:We have modified it. |
| Line 251: would you accurately say the initial low to high moisture content reduction was due to evaporation and the gradual decrease after 24h due to diffusion? | Line 264:We have modified it. Deleted ‶The rate of moisture content reduction moved from low to high, and it gradually decreased after 24h.″ |
| Line 258: increase in the mass fraction of what? | Line 271:We have modified it.“in the mass fraction of free water at the surface” |
| Line 264: “was smaller”  “became slower”? | Line 277: We have modified it. |
| Line 265: “evaporation surface area.” | Line 278-279: We have modified it. |
| Line 266-267: not a sentence. Confusing, edit for clarity. | Line 279-282:We have modified it. The heat transfer of air from the surface of oil sludge to the interior of oil sludge can realize convective heat transfer between hot air and oil sludge. When the free water inside the oily sludge is heated, it gradually migrates to the surface and diffuses into the air. |
| Line 268: Raising  Increasing | Line 282:We have modified it. |
| Line 270: “required semi-dried”  “required to be semi-dry” | Line 284:We have modified it. |
| Line 271: “temperature should be maintaining at greater than 10°C” | Line 285:We have modified it. |
| Line 271: Figure S2?? | Line 285:We have modified it. |
| Section 274: leachate or emulsion. Choose one. | Line 291:We have modified it.Choose leachate |
| Line 286: over time? | Line 302-303:We have modified it. Replace ‶As the drying process was extended″ with ‶over time″ |
| Line 291: were coating thickness and area of emulsion surface measured? | Line 307:There is no measurement, here is a qualitative analysis. By measuring the changes in surface tension, the hydrophilic and lipophilic properties of different forms of water can be analyzed without further quantitative analysis. |
| Line 297-300: great text for figure 2 caption | Line 311-314:We have modified it. |
| Line 302: Figure 2b?? | Line 316: We have modified Fig 5b to Fig 2b |
| Line 303: leaching solution →leachate | Line 317:We have modified it. |
| Line 304: situation → observations | Line 318:We have modified it. |
| Line 316: what are double layers? | Line 329-330:We have modified it. ‶electric double layers″ |
| Line 320: what is “It”? The surfactant? | Line 333:We have modified it. ‶The surfactant″ |
| Line 321: electricity negatively charged species?? | Line 334:We have modified it. ‶negatively charged species″ |
| Line 324: define DSC | Line 337:We have modified it. |
| Line 325: “…was reduced by 69% after drying for 10 min at 100°C (Fig. 4).” | Line338:We have modified it. |
| Line 344: missing “free water”? and combining of sentences. “…the interior free water of the oil sludge quickly increased resulting in increased evaporation (e to f).” | Line 356-357:We have modified it. |
| Line 330-331: delete “(a,b,c,d,e,…for the evaporation rate)” and place in caption (already there). | Line 343:We have modified it. |
| Line 331-332: “the rate was reduced between b and c as remaining free water is evaporated” | Line 343-344:We have modified it. |
| Line 333-334:”oily sludge was demulsified and the free water increased over a short time leading to an  increase in the rate of mass loss (c to d phase). | Line 345-346:We have modified it. |
| Line 334-336: “As the emulsified water decreased, the heat was …to the interior, and the bound water…” | Line 346-347:We have modified it. |
| Line 336: “continuously reduced”  “decreased” | Line 348:We have modified it. |
| Line 347: emulsion evaporated? New sentence: Most of the …? Rewrite. | Line 358-359: We have modified it. ‶The water in the form of bound water between internal pores of oily sludge particles and all free water evaporated. ″ |
| Line 350: “were shown in”  “are shown in” | Line 361:We have modified it. |
| Line 352: delete significantly | Line 363:We have modified it. |
| Line 353: edit – “…for the non-amended oily sludge…” | Line 364:We have modified it. |
| Line 355: “four stages to the drying processes as outlined below.” These four stages are not clearly shown in figure 5. Describe how these are shown in the figure. | Line 365:We have modified it. ‶The experimental process and the TGA curves show the stages to the drying process.″ |
| Line 359-360: confusing. Edit for clarity. | Line 368-371:We have modified it. ‶When the surface of oily sludge is heated, the free water on the surface begins to evaporate. After oily sludge sample was added to the OSM, the oily sludge sample first reacted with free water due to water absorption from the powdered material. The water in the emulsion form was gradually converted into free water. The emulsified water was transformed into free water form. ″ |
| Line 396: unbold From | Line 405: We have modified it. |
| Line 398: delete “the” | Line 417: We have modified it. |
| Line 414: best effect = most important effect? – how was this determined? | Line 423: We have modified it. Chose ‶most important effect″ |
| Line 421-425: accidental sentence? Reread and edit. | Line 430-432: We have deleted it. |
| Line 425-428: “At environmental temperatures exceeding 10°C the OSM significantly shortened the  natural semi-drying time of the oily sludge from ___ to __” | Line 432-434:We have modified it. |
| Line 432: facilitating | Line 438:We have modified it. |
| Line 433: speeding up | Line 439:We have modified it. |
